# Turing Patterns of Non-linear S-I Model on Random and Real-Structure Networks with Diarrhea Data

**DOI:** 10.1038/s41598-019-45069-3

**Published:** 2019-06-20

**Authors:** Prama Setia Putra, Hadi Susanto, Nuning Nuraini

**Affiliations:** 1 Industrial and Financial Mathematics Research Group, Department of Mathematics, Institut Teknologi Bandung, Ganesha 10, Bandung, 40132 Indonesia; 2 Department of Mathematical Sciences, University of Essex, Wivenhoe Park, Colchester, CO4 3SQ United Kingdom

**Keywords:** Applied mathematics, Infectious diseases

## Abstract

Most developed models for solving problems in epidemiology use deterministic approach. To cover the lack of spatial sense in the method, one uses statistical modeling, reaction-diffusion in continuous medium, or multi-patch model to depict epidemic activities in several connected locations. Here, we show that an epidemic model that is set as an organized system on networks can yield Turing patterns and other interesting behaviors that are sensitive to the initial conditions. The formed patterns can be used to determine the epidemic arrival time, its first peak occurrence and the peak duration. These epidemic quantities are beneficial to identify contribution of a disease source node to the others. Using a real structure network, the system also exhibits a comparable disease spread pattern of Diarrhea in Jakarta.

## Introduction

Kermack and McKendrick^1^ made a seminal contribution in understanding the spreading of epidemics by introducing a mathematical model now known as the Susceptible-Infected-Recovered (S-I-R) model where limited populations with an epidemic are assumed to be divided into three compartments. Individuals in the susceptible compartment could move into the infected one with a certain transmission rate from interacting with contagious individuals. After some times, the contagious individuals would recover and hence moved into the recovered compartment. Beyond the peak of the epidemic, the model showed that the epidemic would decrease until all of the contagious individuals were moved from the infected into the recovered compartment, i.e. the epidemic extincts.

The compartmental model was further developed by Diekmann *et al*.^2^ by introducing an important parameter related to the infection in the virgin population, which is now known as the basic reproduction ratio. The parameter can be obtained from the spectral radius of a representative matrix called as the next generation matrix. van den Driessche and Watmough^3^ explained the matrix as that of expected new infections in one compartment to another compartment. The ratio is important because it can give the information about the effect of transmission rate towards the infection in the population. Recent improvements to the S-I-R model include the consideration of direct transmission diseases from human to human (e.g., chickenpox, measles, and whooping cough)^4^, the prevention strategies such as immunization^4^, vectorial transmitted disease (also known as host-vector model)^4^, and internal transmission^5^ and immune system^6^ for a particular disease.

All of the developed models mentioned above were built in a temporal sense, i.e. they only explain the epidemic in a particular area. To tackle spatial lackness in compartmental approach, a mathematical model to simulate disease spread was developed by including human movement^7^. Besides simulations, a map of disease spreading was often used to perform risk analysis using certain index^8–12^. However, to make a proper calculation, complex datasets might be required in order to obtain the best models that explain the natural observation. Another approach is through pattern formations based on the reaction-diffusion systems, i.e. spatial dependence of the epidemic and populations^13–17^, or pattern detections based on the wave formation^18^.

Following the report on pattern formation in chemical reactions^19^, Turing explained the emergence of pattern to be driven by the diffusion in the system that changed the stability of the equilibria^20^. In epidemic modeling using reaction-diffusion systems, pattern could be obtained from the equilibrium point in the endemic state. The idea was inspired by Fisher’s equation, which explained the dispersion of organism using the logistic equation^21^. Cruickshank^13^ then adopted the logistic equation as the recruitment rate for the susceptible population in the S-I model. However, the dispersion of the organism only occurs for the contagious individual. It was then extended by considering the diffusion on the susceptible individuals as well^14,15^, cross-diffusion events (that involve the motion of the susceptible individuals because of the presence of contagious ones)^16,17,22^ and the basic S-I-R model of Kermack and McKendrick^1^ in a reaction-diffusion system^23^. The latter type of extensions shows the different kinds of spatial patterns in the spread of diseases^24^ with transitions as an emergent property that can serve as a potential trend indicator^25^.

Following the inception of network studies that were inspired by the large structure of internet connections^26–29^, epidemic models with network connection to incorporate the spatial dependence were then introduced^28–31^. In this case, each node on the network represents an individual in the population and its state can change from healthy into infected depending on the connection to infected individuals with some transmission rate^30^. An epidemic model on networks using three compartments was then developed, where in this case each node represents a group of population containing susceptible, infected, and recovered individuals^31^. The model could explain the phenomenon that epidemic cases depend on social topology and may rise when an epidemic attacks individuals with high connectivities^31^. How human behavioural and social dynamics affects disease spreading and prevention in well-mixed and networked populations have also been widely studied^32^.

A different perspective was introduced by considering each node as an area and disease transmission through the population occurs in that area^28,29^. Connections between nodes, i.e. edges, represent the motion of people between the areas. This model was developed to capture disease transmission phenomena in metapopulation events^28^, that can explain the influence of connections through their coupling strength^29^. It was shown that disease transmissions on Winnipeg city and its smaller neighborhood cities could be driven by a small city with low coupling strength. Modelling in metapopulation or networks can give an understanding the effects of people’s motions in disease spreading. However, recognizing disease spread pattern as the mobility consequence is challenging since it can involve many factors such as motion trajectories and directions between some locations, pattern of people movement which can represent commuter or migration activity and how fast people move from one area to another. Recently, the study on pattern formation through reaction-diffusion model arose in network organized systems^33^. The network organized reaction-diffusion model exhibits patterns which represent movement structurally easier to understand than continuous model. We may be difficult to identify the behavior of continuously area since the original reaction-diffusion model represents population density on every point. In fact, people are occupying places discretely and continuous model may be not appropriate in disease spread case. Developing model of a big region into some points offers model relevance to real problem and clearer interpretation. Therefore, exploring disease spread using reaction-diffusion model in network-organized structure is considerably more appropriate.

In this work, we investigate the possibilities of emerging patterns from the reaction-diffusion of an S-I model in networks. We consider a two compartments epidemic model with logistic growth and a not constant total population. Numerical simulations have been done to reveal the complex patterns in the S-I model that depends on diffusion coefficient parameters.

Analysis of pattern emergence presented in this paper uses a mean-field approximation, visualizing the stationary solution in the average sense^33–35^. Even though the diffusion coefficient is set below threshold, pattern formation can occur and will be shown through hysteresis analysis. It emerges because of an initial condition selection. By varying the initial condition of certain nodes and set the other nodes near a homogeneous equilibrium, we present simulations of differentiated nodes along with the analysis to compute their values at steady states.

Our model represents a situation where locations with similar characteristics show a variation in their disease spread patterns. The important findings of the developed model are epidemic arrival time pattern of an emerging infected node and the occurrence of first epidemic peak and its duration. Node degrees and mobility rate drive the disease to distribute broadly. We present epidemic diarrhea data of Jakarta and show that the pattern is qualitatively comparable with reaction-diffusion model.

The outline of the paper is given as follows. We start with introducing the model. Next, we discuss the system’s equilibria and study their stability, which provide preceding information to pattern formation. Numerical simulations for moderate and large random networks are then performed. The S-I model shows the occurrence of hysteresis when the ratio of the diffusion coefficients between the populations is set below a critical value. A single-differentiated-node analysis is performed to understand the numerical observation. Finally, a real network structure representing districts of Jakarta is considered and epidemic diarrhea data shows a comparable pattern with the numerical results.

## Epidemic Model in Networks

### Full model

The reaction-diffusion systems in networks^33,36,37^ are described generally by

1 $$\begin{array}{rcl}\frac{d}{dt}{u}_{i}(t) & = & f({u}_{i},{v}_{i})+{D}_{u}\sum _{j=1}^{N}\,{L}_{ij}{u}_{j},\\ \frac{d}{dt}{v}_{i}(t) & = & g({u}_{i},{v}_{i})+{D}_{v}\sum _{j=1}^{N}\,{L}_{ij}{v}_{j},\end{array}$$

where *u*_*i*_(*t*) and *v*_*i*_(*t*) are local densities of the activator and inhibitor species at node *i* and time *t*. The kinetic functions on the *i* th node are given by *f*(*u*_*i*_, *v*_*i*_) and *g*(*u*_*i*_, *v*_*i*_). The diffusion process in the system is going through the network, whose topology is defined by *N* number of nodes and represented by *N* × *N* adjacency matrix *A* with the element *A*_*ij*_ equal to 1 if there is a link between *i* th node and *j *th node and 0 if there is no link between them. The degree of node *i* is given by ${k}_{i}={\sum }_{j\mathrm{=1}}^{N}\,{A}_{ij}$. The Laplacian matrix *L* whose element is given by *L*_*ij*_ = *A*_*ij*_ − *k*_*i*_*δ*_*ij*_ where *δ*_*ij*_ is Kronecker’s delta that is equal to 1 when *i* = *j* and 0 when *i* ≠ *j*. *D*_*u*_ and *D*_*v*_ act as the diffusion coefficients of species *u* and *v*, respectively. The coefficients *D*_*u*_ and *D*_*v*_ are then scaled to *D*_*v*_/*D*_*u*_ = *σ* and *D*_*u*_ = *ε*.

As a particularly interesting case of (1), here we consider a network of the nonlinear S-I model

2 $$\begin{array}{rcl}\frac{d}{dt}{S}_{i}(t) & = & f({S}_{i},{I}_{i})+\varepsilon \sum _{j=1}^{N}\,{L}_{ij}{S}_{j},\\ \frac{d}{dt}{I}_{i}(t) & = & g({S}_{i},{I}_{i})+\sigma \varepsilon \sum _{j=1}^{N}\,{L}_{ij}{I}_{j},\end{array}$$

where

3 $$f(S,I)=rS(1-\frac{S}{K})-\beta \frac{SI}{S+I},\,g(S,I)=\beta \frac{SI}{S+I}-\gamma I,$$

*S*_*i*_(*t*) and *I*_*i*_(*t*) are local densities of susceptible and infected individuals, respectively, in *i* th node at time *t*. The susceptible and the infected individuals are analogous to the activator and inhibitor species in system (1), where population increment happens in the susceptible compartment as disease can inhibit the growth of the population. Parameter *r* is the growth rate of the susceptible population. Parameters *K*, *β*, and *γ* are the carrying capacity, the transmission rate, and the mortality rate caused by the disease, respectively. The form of the kinetic functions *f* and *g* is due to Cruickshank *et al*.^13^ and Sun *et al*.^22^ with the logistic-like equation as the recruitment rate^13,14,17,22^, which usually is assumed to be constant. In this case, the transmission process occurs between susceptible and a proportion of infected, i.e. not the total, population. The interaction that occurs in the model represents a disease transmission between locations. Herein, the networks are assumed to be scale-free.

### Instability of the uniform equilibria

Without diffusion in (2), i.e. *ε* = 0, one can easily identify that the remaining system has two equilibria. They are the disease free equilibrium *E*_1_ = (S^*^, I^*^) with S^*^ = *K* and I^*^ = 0, and the endemic equilibrium *E*_2_ = (S^*^, I^*^) with

4 $${S}^{\ast }=\frac{K(r+\gamma -\beta )}{r},\,{I}^{\ast }=\frac{K(r+\gamma -\beta )(\beta -\gamma )}{r\gamma },$$

provided that

5 $$\gamma  < \beta  < \gamma +r,$$

to be biologically relevant (as outside the interval, I^*^ < 0).

Starting from the neighborhood of *E*_1_, the system will allow the susceptible to increase its population until it reaches the carrying capacity, i.e. the equilibrium is unstable. On the other hand, the endemic equilibrium *E*_2_ preserves the disease to stay and infects the population for a long time, i.e. the equilibrium is stable (see Methods section).

**Figure 1:**
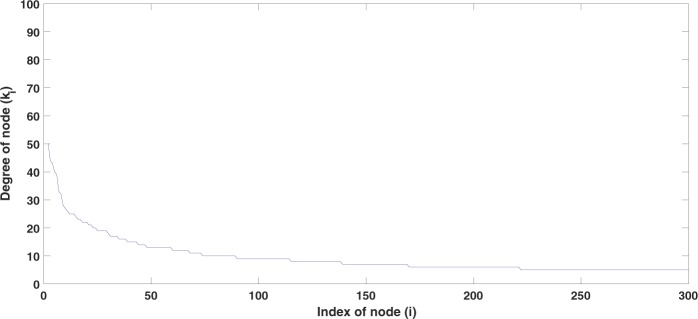
Distribution of the node degrees in our simulation. Here, *N* = 300 nodes and mean degree 〈*k*〉 = 10.

Now we consider the S-I model (2) with diffusion. We consider a scale-free network with a size of *N* = 300 and mean degree 〈*k*〉 = 10. The parameters are set to *r* = 0.27, *K* = 1000, *β* = 0.5, and *γ* = 0.25. The distribution of the node degrees of the network is shown in Fig. 1. We sort the nodes in the order of their degrees where index 1 refers to the node with the largest node degree and index *N* to that with the smallest node degree.

To exhibit patterns via Turing bifurcation^38^, the critical eigenvalues of the linearisation operator around an equilibrium of the model must be complex valued with positive real part. The diffusion process can only change the stability of the endemic equilibrium *E*_2_, not the disease free *E*_1_ (see Methods section). Hence, below we only consider the former equilibrium. From the linearized equations, we obtain that to obtain unstable eigenvalues, the parameters need to satisfy the condition (*β*^2^ − *rβ* − *γ*^2^)/*β* > 0.

**Figure 2:**
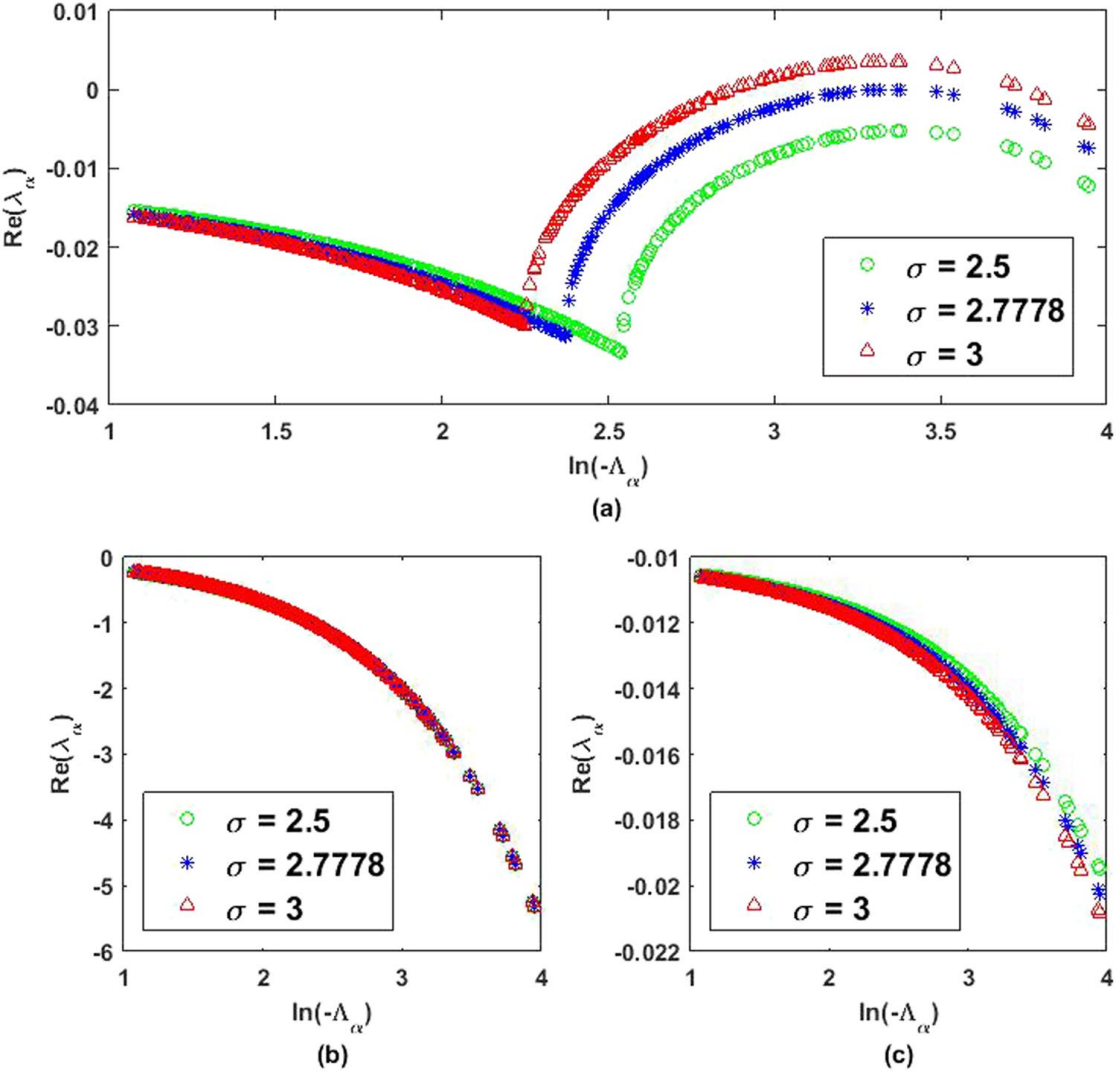
Eigenvalues *λ*_*α*_ of the linearised operator of (2) around the endemic equilibrium for *α* = 1, 2, …, *N* on the scale-free network in Fig. 1 are plotted as a function of the Laplacian eigenvalues Λ_*α*_. The parameters are *r* = 0.27, *K* = 1000, *β* = 0.5, and *γ* = 0.25 with (**a**) *ε* = 0.00105 (**b**) *ε* = 0.000105 (**c**) *ε* = 0.105.

The eigenvalues are depicted in Fig. 2 corresponding to three different values of *σ*. From the plots, we can see that Turing bifurcation shall occur when *σ* is above the critical value *σ*_*c*_ = 2.77778. In this case, patterns will emerge in the system. However, for a fixed number of nodes *N*, the coupling strength *ε*, which is the diffusion coefficient of susceptible compartment, will play an important role. From Fig. 2b,c, Turing instability does not occur even though parameter *σ* has been set larger than the critical value. The coupling strength between the nodes *ε* cannot be too small nor too large to obtain the partition showing Turing instability. Larger size of network is necessary to yield Turing instability in Fig. 2b. While that in Fig. 2c shows declining real part of the eigenvalues. We can conclude that we may not always obtain Turing instability although the critical parameter has been met.

The critical value *σ*_*c*_ can be obtained analytically from the characteristics polynomial equation *λ*^2^ − [*f*_*S*_ + *g*_*I*_ − (1 + *σ*)*θ*]*λ* + [(*f*_*S*_ − *θ*)(*g*_*I*_ − *σθ*) − *f*_*I*_*g*_*S*_] = 0, where *θ* = − *ε*Λ (see Methods section). The critical parameters *σ*_*c*_ and *θ*_*c*_ correspond to the condition *λ*(*θ*_*c*_) = *λ*′(*θ*_*c*_) = 0 and *λ*′′(*θ*_*c*_) < 0 where prime is the derivation respects to *θ*. Thus, the critical parameters are given by

6 $${\sigma }_{c}=\frac{1}{{f}_{S}^{2}}({f}_{S}{g}_{I}-2{f}_{I}{g}_{S}+2\sqrt{{f}_{I}{g}_{S}({f}_{I}{g}_{S}-{f}_{S}{g}_{I})}),\,{\theta }_{c}=\frac{{g}_{I}+{\sigma }_{c}{f}_{S}}{2{\sigma }_{c}}.$$

The expression will yield *σ*_*c*_ = 2.77778 for *r* = 0.27, *K* = 1000, *β* = 0.5, and *γ* = 0.25, which is in agreement with the numerical observation.

### Pattern emergence in the S-I model

**Figure 3:**
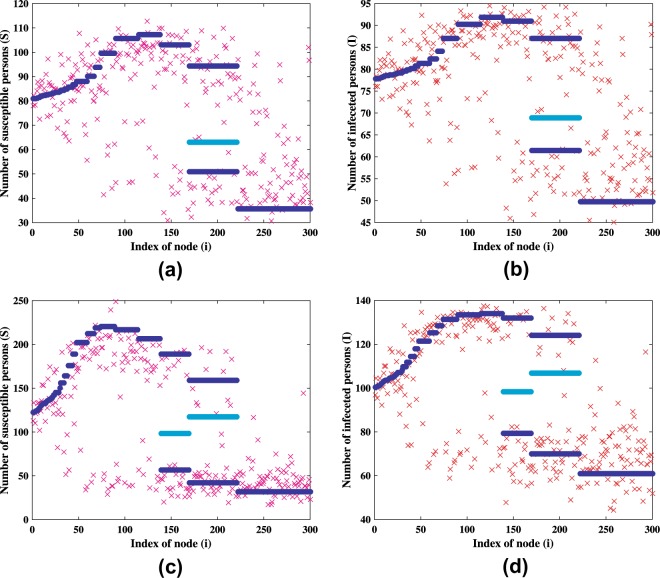
Stationary patterns of system (2) obtained numerically. The stationary pattern for (**a**) susceptible and (**b**) infected nodes using *σ* = 3, and for (**c**) susceptible and (**d**) infected nodes using *σ* = 5. The other parameters are the same as those in Fig. 2 and *ε* = 0.005. Solutions of the mean-field system (7) using the same parameters are also plotted with (**a**) *H*^(*S*)^ = 79.8644, (**b**) *H*^(*I*)^ = 77.2836, (**c**) *H*^(*S*)^ = 116.815, and (**d**) *H*^(*I*)^ = 98.4894.

For the network organization shown in Fig. 1, even though we set the parameters to be the same for all the nodes, the diffusion process can make differences for the stationary solution of system (2). Using the endemic equilibrium perturbed with a small deviation as the initial condition for the simulation, we show in Fig. 3a,b the stationary solution of system (2) using *σ* = 3 and in Fig. 3c,d using *σ* = 5. Magenta and red nodes depict the stationary solution of susceptible and infected compartments, respectively. As *σ* increases, the stationary solution will generally split into two clusters. However, the nodes with high connectivity will maintain its position near the weighted average of the stationary solution of each compartments.

The behavior of the stationary solution can be explained by the mean-field approximation^33–35^, which is the averaged value of all the nodes. Defining the weighted average of the stationary solution as ${H}^{(S)}={\sum }_{i=1}^{N}\,\frac{{k}_{i}}{{\sum }_{j}{k}_{j}}{S}_{i}$ and ${H}^{(I)}={\sum }_{i=1}^{N}\,\frac{{k}_{i}}{{\sum }_{j}{k}_{j}}{I}_{i}$, as the global mean-field, the mean-field equation is then described by

7 $$\begin{array}{rcl}\frac{d}{dt}{S}_{i}(t) & = & r{S}_{i}(1-\frac{{S}_{i}}{K})-\beta \frac{{S}_{i}{I}_{i}}{{S}_{i}+{I}_{i}}+\varepsilon {k}_{i}({H}^{(S)}-{S}_{i}),\\ \frac{d}{dt}{I}_{i}(t) & = & \beta \frac{{S}_{i}{I}_{i}}{{S}_{i}+{I}_{i}}-\gamma {I}_{i}+\sigma \varepsilon {k}_{i}({H}^{(I)}-{I}_{i}).\end{array}$$

The solutions of the mean-field Eq. (7) are depicted in Fig. 3. Good agreement is clearly seen. The mean-field equations admit two solutions, which are stable and unstable. The stable and the unstable solutions are represented by dark blue line and by the light blue line, respectively.

### Hysteresis of patterns

Stationary solutions of the main Eq. (2) depend on the initial condition. We will show it from a hysteresis phenomenon.

**Figure 4:**
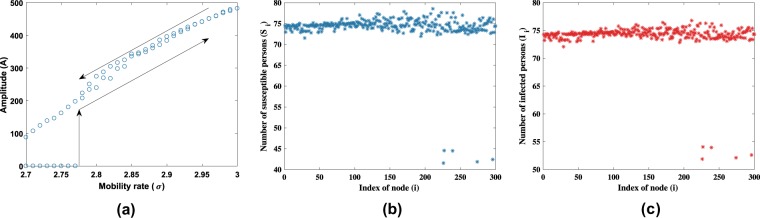
Hysteresis of (2) using parameters in Fig. 2 and *ε* = 0.005. (**a**) The amplitude of Turing pattern in the S-I model. A typical pattern for (**b**) *S* compartment and (**c**) *I* compartment at *σ* = 2.7.

Define the amplitude *A* as the distance of fields in each node from the equilibrium of (2) without diffusion, i.e. $A={[{\sum }_{i=1}^{N}{({S}_{i}-{S}^{\ast })}^{2}+{({I}_{i}-{I}^{\ast })}^{2}]}^{1/2}$. The amplitude *A* is depicted in Fig. 4a.

The presence of a jump from the endemic equilibrium *A* = 0 to non-zero *A* as we increased *σ* is due to Turing instability we discussed earlier, where the endemic equilibrium changes its stability. This occurs at the critical value *σ*_*c*_. Normally, Turing patterns emerge as a pitchfork bifurcation. In the supercritical case, one would expect the nodes to relax to the endemic equilibrium again when parameter *σ* is set below the critical value. However, from Fig. 4a, we observe a hysteresis where this is not the case. Figure 4b shows a non-uniform stationary solution of (2) at *σ* = 2.7, which is below the critical value.

From the figure, we obtain that nodes with high connectivity maintain its position near the equilibrium. Notably we see the presence of isolated nodes with a value that is relatively distinguishable and far from the average value. Such a node is referred to as a single differentiated node (SDN)^36^.

To analyze SDN, we shall assume that the nodes are mostly in the endemic equilibrium except the *i* th one. Then, system (2) can be reduced into

8 $$\begin{array}{rcl}\frac{d}{dt}{S}_{i}(t) & = & r{S}_{i}(1-\frac{{S}_{i}}{K})-\beta \frac{{S}_{i}{I}_{i}}{{S}_{i}+{I}_{i}}+\varepsilon {k}_{i}({S}^{\ast }-{S}_{i}),\\ \frac{d}{dt}{I}_{i}(t) & = & \beta \frac{{S}_{i}{I}_{i}}{{S}_{i}+{I}_{i}}-\gamma {I}_{i}+\sigma \varepsilon {k}_{i}({I}^{\ast }-{I}_{i}),\end{array}$$

where *k*_*i*_ is the node degree.

**Figure 5:**
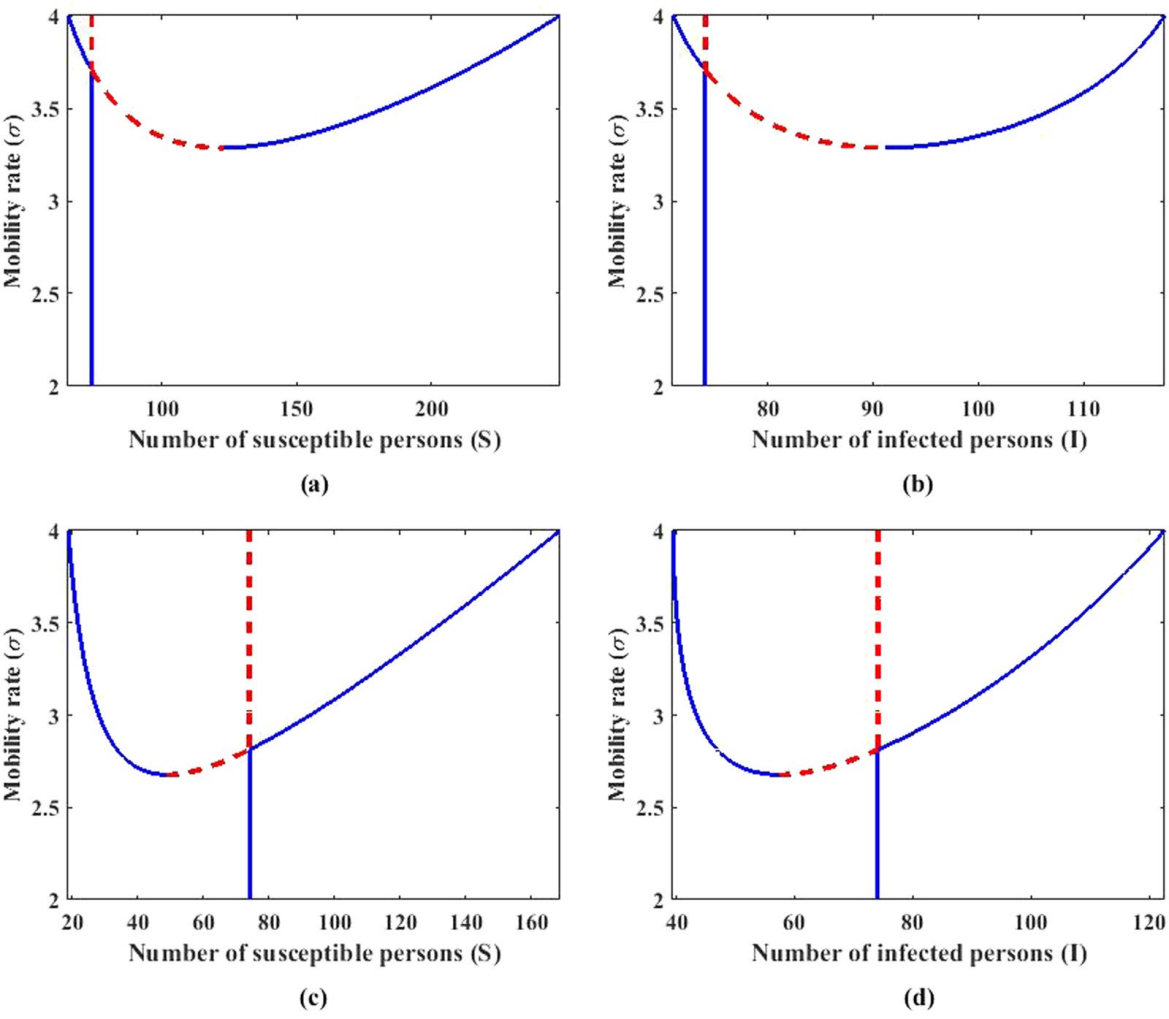
Stationary solutions of (8) using parameters in Fig. 2 with *ε* = 0.005 and node degree *k*_*i*_ = 12 (**a**,**b**) and *k*_*i*_ = 5 (**c**,**d**). Panels (a) and (c) are for the susceptible compartment, while (**b**) and (**d**) are for the infected one. Blue line and red dashed line indicate the stable and unstable solutions of (8), respectively.

Stationary solutions of (8) are depicted in Fig. 5 for two different node degrees *k*_*i*_ = 12 in Fig. 5a,b and *k*_*i*_ = 5 in Fig. 5c,d. The vertical axis is *σ*, while the horizontal one is the dependent variables. In the figures, blue line and red dashed line represent stable and unstable solutions, respectively. The plots show that to obtain an SDN, the higher the node degree, the higher the value of *σ* is required. This can explain the persistence of nodes with high connectivity to maintain their position near the endemic equilibrium, while nodes with lower connectivity can vary quite far from the endemic equilibrium.

**Figure 6:**
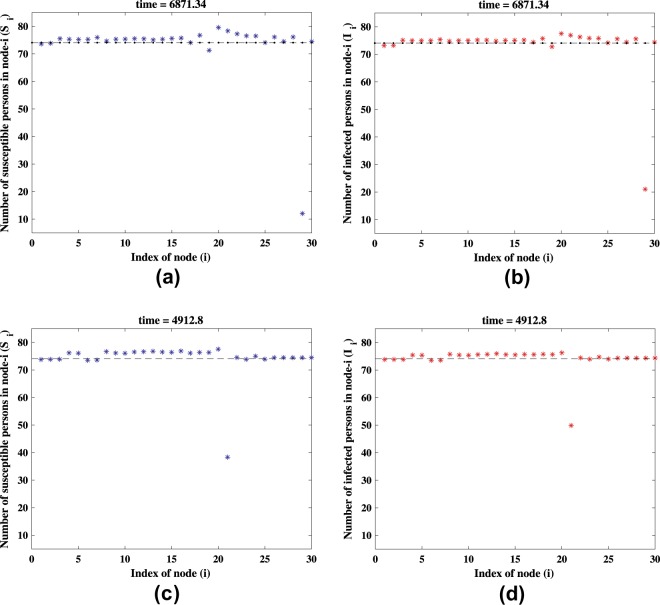
Stationary solution of system (2) using parameters in Fig. 2, *ε* = 0.005, and *σ* = 2.74. Simulation is performed using network with *N* = 30 and degree of node (25, 23, 21, 19, 18, 16, 15, 14, 13, 13, 12, 11, 10, 10, 10, 9, 8, 8, 7, 6, 5, 4, 3, 3, 3, 2, 2, 2, 2, 2). Solution of a node with degree *k*_*i*_ = 2 (**a,b**) *k*_*i*_ = 5 (**c,d**). Panels (**a**) and (**c**) are for the susceptible compartment, while (**b**) and (**d**) are for the infected compartment.

To show that, we have performed several simulations using networks with moderate size (*N* = 30) and carefully selected initial conditions. By initially choosing the predicted stable SDN for the *i* th node and endemic equilibrium for the others, we show the results in Fig. 6, where one can clearly observe the presence of SDN in the network. This hence confirms the observation of non-vanishing amplitude in Fig. 4 when the diffusion ratio *σ* is even below the critical value *σ*_*c*_.

### Effects of parameters on non-uniform states

**Figure 7:**
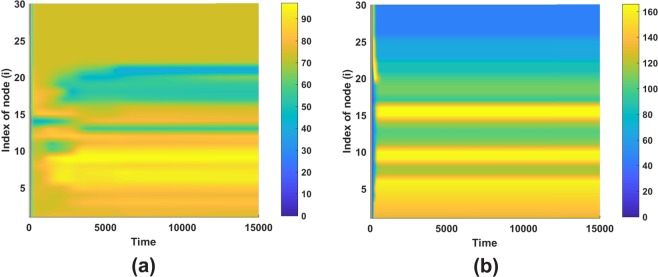
Stationary patterns using parameters in Fig. 2 and *ε* = 0.005 with (**a**) *σ* = 3 and (**b**) *σ* = 10.

Mobility in system (2) is represented by the parameters *ε* and *σ*, which show the movement rate of healthy and infected populations, respectively. The disease spread is affected by the arrival of symptomatic or asymptomatic ill persons at a new destination^39^. While in previous sections, we are interested in the characteristics of stationary patterns, it is important to also study transient dynamics of the system leading to stationary states. For the sake of clarity, we now consider moderate size network. Nodes are ordered based on the size of their connectivity degree. The first and the last index indicate node with the largest and smallest node degree, respectively. Taking different values of *σ*, we show the resulting stationary patterns in Fig. 7. We obtain that the distribution of infected people tends to spread evenly for a smaller *σ*, i.e., low movement rate of infected compartment (Fig. 7a). A larger *σ* (Fig. 7b) makes the infected population concentrate at nodes with moderate and large degree connectivities.

**Figure 8:**
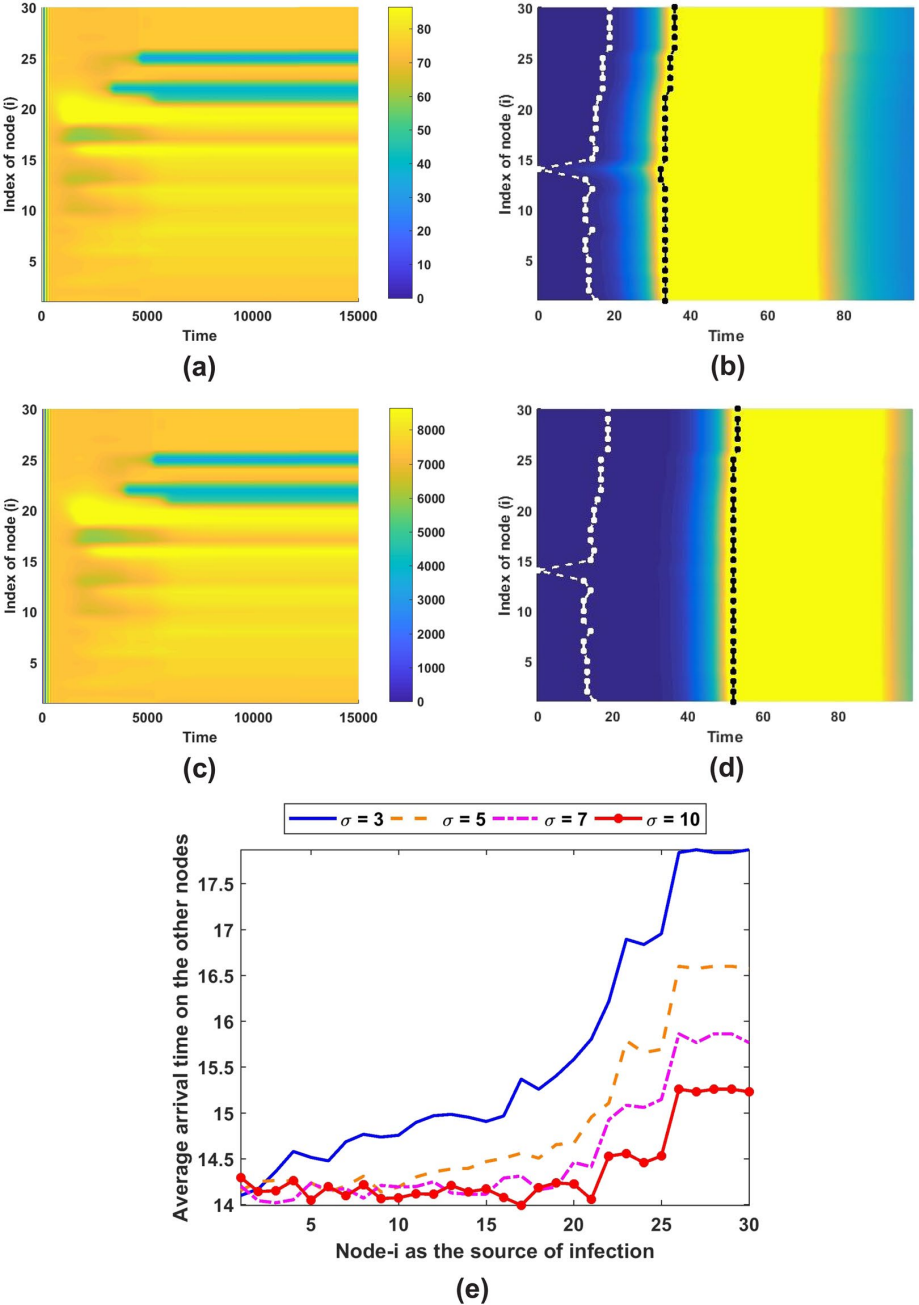
Patterns using parameters in Fig. 2 and *ε* = 0.005 with (**a**) *K* = 1000 and (**c**) *K* = 100000, while (**b**) and (**d**) are first epidemic peak occurrence for pattern in and epidemic arrival time in each nodes depicted by white dot-line while the first epidemic peak depicted by black dot-line. Panel (e) depicts the epidemic arrival time on scale-free networks as a function of source of infection node.

We have investigated system (2) using a moderate size network when initially an infection is introduced at one node. Figure 8 shows the dynamics of the system as the solution evolves in time. It is interesting to note that before reaching a stationary state, the system oscillates.

It is important to note that the resulting pattern is rather independent of the growth, transmission, and death rates. The carrying capacity does not influence the longterm pattern. It only affects the magnitude of the compartments. Nevertheless, those parameters has a significant impact on the so-called first epidemic peak which will be explained in the next sub-section. Considering a larger *K* yields a slower first epidemic peak as illustrated in Fig. 8b,d. This finding shows the linearity between carrying capacity and first peak occurrence, i.e., the disease needs more time to infect large population.

### Epidemic arrival and peak times

The epidemic arrival time can be defined as the time when an infected person is detected on a disease free node. This event can be identified when there is only one node which has been infected while the other nodes are free from the disease and the infected node spreads the disease to the other nodes after that. Source of infection node becomes important in distributing the disease among the nodes. On the average, the disease is quickly distributed when node with large connectivity is infected initially. Degree of node in this study has an equivalent role to the effective distance in Brockmann and Helbing article^40^. In that article, an effective distance can be used to estimate the epidemic arrival time in new locations. Here, the movement rates between nodes are assumed to be equal and the significance of mobility in the model is explained through node connectivity. Besides connection factor, the mobility of an infected person also has a role in accelerating the transmission to a new location (Fig. 8e). A larger movement rate causes nodes with larger connectivity to have a similar arrival time (Fig. 8e). As discussed by Arino and Portet^29^, nodes with high connectivity (small distance with high movements) can be indistinguishable; in our case, the distance is represented by the node degree. It is noted that restraining a sick person to travel to another location becomes important when the disease has a potential to be transmitted easily and globally^39^.

The first epidemic peak is defined as the period when the size of infected population is about 10% of total population in the early time of first infection and back to the same size after a certain duration. Population size shows a little significant impact on the arrival time but it has a connection with peak occurrence. First epidemic peak occurs as the disease emerges on that location. Larger population size requires a longer time to reach its peak. On the other hand, a larger mobility rate delays the peak and reduces its duration to occur although it can increase the size of infected population. Table 1 explains the overall average and deviation for epidemic time of arrival, first epidemic peak occurrence and duration.

**Table 1:** Average and deviation of epidemic time of arrival, first epidemic peak occurrence and duration.

			*σ* = 3	*σ* = 5	*σ* = 7	*σ* = 10
Random network	K = 1000	Epidemic time of arrival	15.6795 ± 1.2932	14.9448 ± 0.9001	14.6425 ± 0.6586	14.4065 ± 0.4478
		First epidemic peak occurrence	32.9124 ± 0.8271	34.6902 ± 0.371	35.1533 ± 0.2839	35.1866 ± 0.1802
		First epidemic peak duration	41.1 ± 0.3497	33.4373 ± 0.3387	31.4712 ± 0.3132	30.4918 ± 0.2521
	K = 1000000	Epidemic time of arrival	15.6122 ± 1.2582	14.9018 ± 0.8894	14.576 ± 0.6467	14.378 ± 0.4201
		First epidemic peak occurrence	50.9367 ± 0.4036	52.8858 ± 0.2847	53.5276 ± 0.379	53.6037 ± 0.2127
		First epidemic peak duration	40.9643 ± 0.4679	33.4728 ± 0.3176	31.3777 ± 0.3008	30.46 ± 0.3056
Real-structure network	K = 1000	Epidemic time of arrival	23.8676 ± 2.8757	20.5564 ± 2.4989	18.6327 ± 2.302	16.788 ± 2.1385
		First epidemic peak occurrence	40.1957 ± 2.826	38.0124 ± 2.5785	36.3736 ± 2.5032	34.5536 ± 2.4423
		First epidemic peak duration	34.7023 ± 0.1321	30.3961 ± 0.1275	29.4102 ± 0.4228	28.1745 ± 0.6393
	K = 1000000	Epidemic time of arrival	23.7889 ± 2.8634	20.5011 ± 2.4918	18.5899 ± 2.3004	16.7497 ± 2.1405
		First epidemic peak occurrence	55.9879 ± 2.8037	53.8482 ± 2.6722	51.8695 ± 2.6561	49.3418 ± 2.5759
		First epidemic peak duration	34.1391 ± 0.3385	30.1298 ± 0.1153	28.6639 ± 0.076	26.7701 ± 0.0631

### Real-structure network patterns and exploration using real data

**Figure 9:**
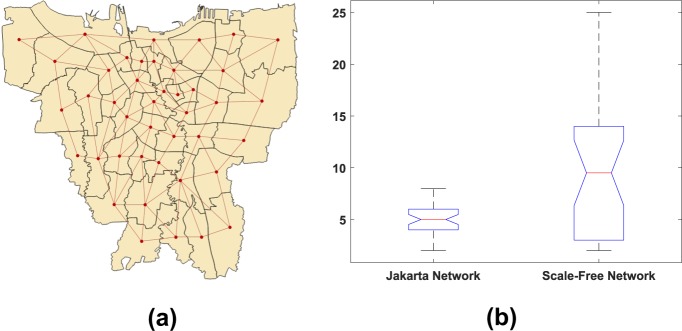
Panel (a) shows the connectivity between districts in Jakarta. Panel (b) shows comparison of degree of nodes between Jakarta network and moderate scale-free network.

In this paper, we also consider a real network structure based on a map. Here, we have chosen Jakarta as our study area to explore disease occurrence. We create a network from the map by connecting every two districts that are adjacent to each other. Practically we examine that condition by finding an edge to overlap between the two districts. The connection between districts in Jakarta is depicted in Fig. 9a where we obtain a network with 42 nodes.

**Figure 10:**
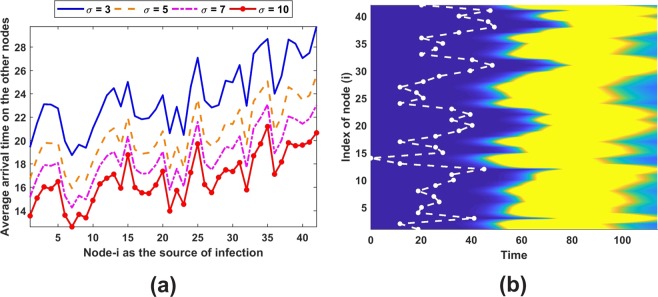
Epidemic pattern on our real-structure network. Panel (a) depicts the graph of epidemic arrival time as a function of source infection node. Panel (b) Shows the epidemic time of arrival and first epidemic peak occurrence pattern.

Our real-structure network has different distribution of node connectivity from scale-free networks considered. Figure 9b depicts the degree of node in Jakarta network that are less vary than scale-free network. Each node node in our real-structure network has at least two and no more than eight connections. On the other hand, we can find some nodes with relatively very large connectivity in scale-free network. Our network generally has similar simulation results compared to scale-free network. Although the epidemic time of arrival has an increasing trend, nodes with larger connectivity do not necessarily accelerate the emergence of disease in new locations (Fig. 10a).

**Figure 11:**
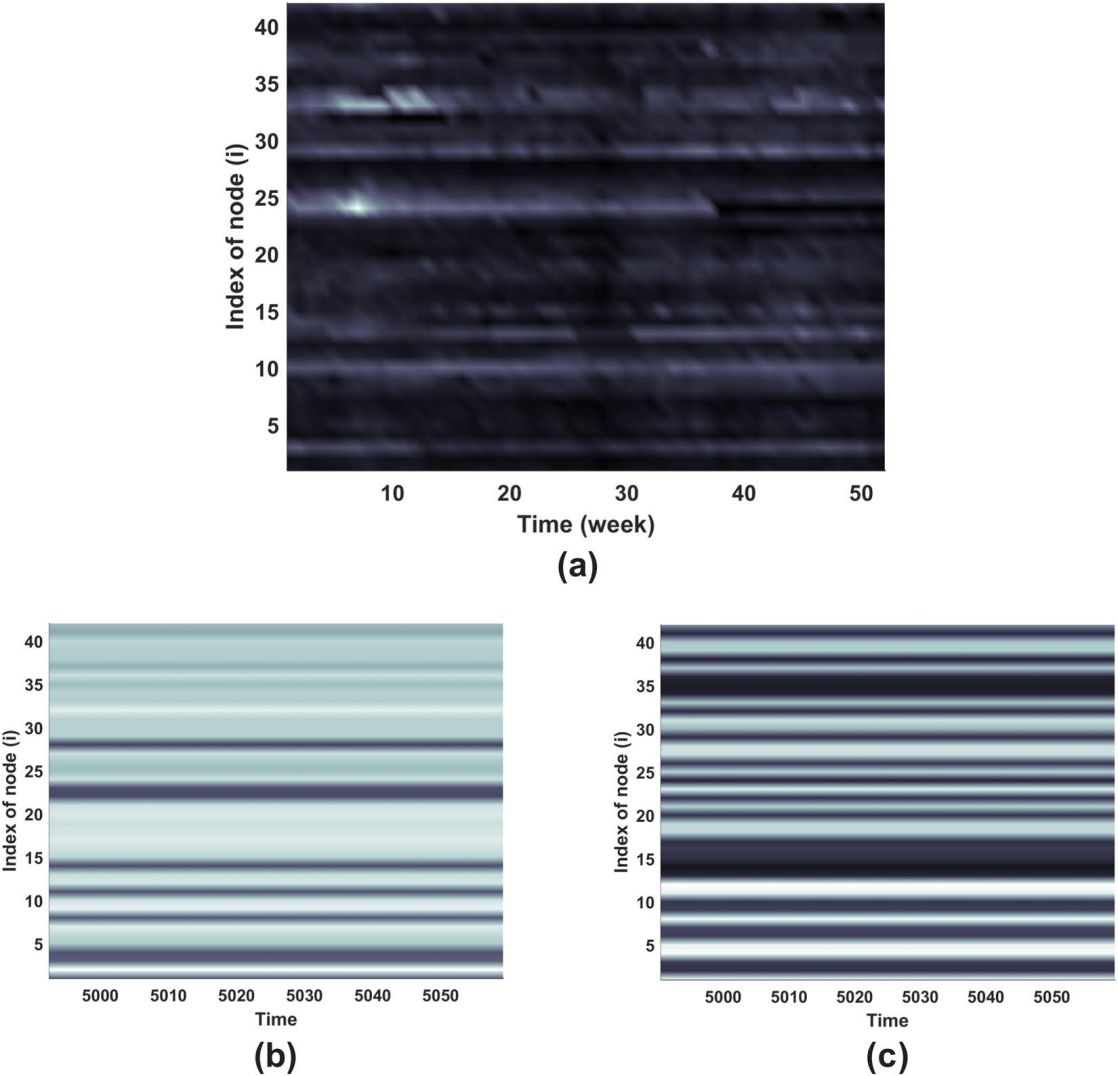
Comparison of diarrhea pattern in a year and resulting patterns from model. Panel (**a**) shows diarrhea pattern in Jakarta in 2015. Stasionary patterns using parameters in Fig. 2 and *ε* = 0.005 with *K* = 242332, (**b**) *σ* = 3 and (**c**) *σ* = 5. The initial conditions to perform the pattens in panel (b) and (c) are selected from 14th node as the infection source while the other nodes are disease free.

Using data obtained from surveilans-dinkesdki.net^41^, we compare a real epidemic condition in Jakarta with theory in this paper. Diarrhea is an example which disease transmission between several locations can be considered as reaction-diffusion process on network-organized system. Figure 11a shows diarrhea cases in 2015 for every districts. The pattern shows that the disease occurs in a year. We simulate model (2) using parameters in Fig. 2 with carrying capacity *K* = 242332. The population in Jakarta in 2015 was around 10 million based on jakarta.bps.go.id^42^. Thus, the total population is divided by 42 districts and yielding around 242,332. We assume the carrying capacity for each districts is the same to reduce the complexity. We use *σ* = 3 and *σ* = 5 to compare the resulted patterns to diarrhea pattern in Jakarta. Stationary patterns from the model with *σ* = 5 in Fig. 11b,c exhibits a comparable pattern to the diarrhea pattern in Jakarta even though the magnitude of simulation result cannot be compared. Initial conditions for the model are selected from perturbed endemic equilibrium of system (3) and one infected in one node while the other nodes are disease free.

Nodes which indicate high cases of diarrhea (average value larger than third quartile of 42 nodes average cases) are 3rd, 9th, 10th, 13th, 24th, 25th, 29th, 33rd, 34th and 42nd node. Simulations with 43 various of initial conditions have been explored to find nodes which indicate high infection. All nodes are classified into four class to see the potential to have high cases of disease. Class of high, medium-high, medium-low and low indicate nodes which occur very often (more than 16 times), often (between 8 and 16 times), rarely (between 3 and 8 times) and very rarely (below 3 times) with high cases in simulation, respectively. The classification result is showed in Table 2. Although the network model shows different nodes of high infection compared with real data, we can learn from this model that nodes with connection number near to average degree of our real-structure network are more likely to show high infection. Nodes with lower or higher degree exhibit to be more difficult to classified as nodes with high infection. More adjustment on the network, such as includes weight on the adjacency matrix to represent real situation on each node, is necessary to perform better result to compare with real data.

**Table 2:** Classification of Nodes to be categorized as nodes indicate high infection.

Classification	Node number (Node degree)
High	2(7); 15(5); 17(5); 18(5); 23(5); 26(4); 18(4); 29(4); 30(4); 31(4)
Medium-high	1(8); 6(6); 9(6); 11(6); 12(6); 13(5); 16(5); 19(5); 22(5); 28(4)
Medium-low	4(6); 5(6); 7(6); 8(6); 14(5); 20(5); 21(5); 24(4); 32(4); 33(3); 34(3)
Low	3(7); 10(6); 27(4); 35(3); 36(3); 37(3); 38(3); 39(3); 40(3); 41(3); 42(2)

## Conclusion

Studying disease transmission using reaction-diffusion in network-organized system shows different perspective in understanding epidemic patterns through deterministic mathematical model. Well-known differential equations to study disease spread is limited to temporal sense explanation on one site. By the time goes on, the epidemic dynamics cannot be restricted to a certain place. The incidence which occurs in one site propagates to different sites. One possibility which can create this condition is human mobility. Further studies in disease spreads reveal the involvement of human mobility in broad infection^28,29,39,43,44^, the mobility can be considered as a diffusion-like process in a fixed structure network.

Our results show that the endemic equilibrium can yield patterns by tuning the mobility parameter of the infected compartment above a critical value. However, Turing instability cannot emerge when mobility for susceptible compartment is wrongly selected. The stationary behavior of the system on the long run can be estimated by studying a mean-field approximation. On the other hand, time independent solution can be different from the mean-field sense. Changes of pattern from the average are shown by hysteresis and SDN events by selecting certain initial conditions. Mobility rate *σ* and carrying capacity *K* determine the infected people distribution among the nodes. Bigger value of the rate creates the infected population to be accumulated on several nodes. Meanwhile, carrying capacity only affects number of victims, but different magnitudes show similar patterns. Extending the simulations by applying only one node as the infection source shows disease propagation time and its period. The arrival time is identified once the disease reaches in a node. Disease early occurrence is strongly affected by mobility rate *σ* and degree of nodes. Higher people movements will quicken the spread while coupling number can show the impact of source node to the other nodes. After the first occurrence propagates, it will create its first peak which be more affected by carrying capacity.

The structure of network has important role in revealing the epidemic and its behavior^40,43,44^. Networks established from real-structure arrangement of Jakarta map also is considered in this study. In general, the network does not show particular different in pattern formation, but cannot exhibit similar characteristic on epidemic time to scale-free network. Different properties of network might create distinct phenomena^40^. However, applying real-structure network on system (2) shows comparable pattern to real data by tuning parameter *σ* properly. This finding creates bigger opportunities to compare reaction-diffusion model in networks to real data and learn disease pattern more widely.

As in this paper we showed the appearance of Turing patterns in the model numerically, it is important to analyse them analytically. It can be done by deriving an amplitude equation using a multiple scale expansion method^45–47^, that can be used to reveal various patterns in the model systematically^45^. This is addressed for future work. Additionally, our work on the effects of directed network topology, that has been shown to significantly change the rule of instability occurrence^37^, in the S-I model will be reported elsewhere.

## Methods

### Stability condition of endemic equilibrium

The stability of the endemic equilibrium is determined from the Jacobian of system (2)–(3), which is the matrix

9 $$(\begin{array}{cc}{f}_{S} & {f}_{I}\\ {g}_{S} & {g}_{I}\end{array})=\frac{1}{\beta }(\begin{array}{cc}{\beta }^{2}-r\beta -{\gamma }^{2} & -{\gamma }^{2}\\ {\beta }^{2}-2\beta \gamma +{\gamma }^{2} & -\gamma (\beta -\gamma )\end{array}),$$

that yields the polynomial characteristics *λ*^2^ + (*γ* + *r* − *β*)*λ* + (*β* − *γ*)(*γ* + *r* − *β*)/*β* = 0. Through the Routh-Hurwitz stability criterion, the endemic equilibrium is stable when (*γ* + *r* − *β*) > 0 and (*β* − *γ*)(*γ* + *r* − *β*)/*β* > 0. This implies that for stability parameter *β* needs to be in the interval *γ* < *β* < *γ* + *r*.

### Stability of network model

Introducing small perturbations to the equilibrium state (S^*^, I^*^), i.e. (*S*_*i*_, *I*_*i*_) = (S^*^, I^*^) + (*δS*_*i*_, *δI*_*i*_), and substituting them into (2) yield

10 $$\begin{array}{rcl}\frac{d}{dt}\delta {S}_{i}(t) & = & {f}_{S}({S}^{\ast },{I}^{\ast })\delta {S}_{i}+{f}_{I}({S}^{\ast },{I}^{\ast })\delta {I}_{i}+\varepsilon \sum _{j=1}^{N}\,{L}_{ij}\delta {S}_{j}\\ \frac{d}{dt}\delta {I}_{i}(t) & = & {g}_{S}({S}^{\ast },{I}^{\ast })\delta {S}_{i}+{g}_{I}({S}^{\ast },{I}^{\ast })\delta {I}_{i}+\sigma \varepsilon \sum _{j=1}^{N}\,{L}_{ij}\delta {I}_{j}.\end{array}$$

We expand the perturbations over the Laplacian eigenvectors,

$$\delta {S}_{i}(t)=\sum _{\alpha =1}^{N}\,{p}_{\alpha }\,\exp \,[{\lambda }_{\alpha }t]{\varphi }_{i}^{(\alpha )},\,\delta {I}_{i}(t)=\sum _{\alpha =1}^{N}\,{q}_{\alpha }\,\exp \,[{\lambda }_{\alpha }t]{\varphi }_{i}^{(\alpha )},$$

where ${\varphi }_{i}^{(\alpha )}$ denotes the *i* th eigenvector of eigenvalue Λ_*α*_, with Λ_*α*_ being an eigenvalue of the Laplacian matrix *L*. Substituting the perturbations into Eq. (10) will result in the eigenvalue problem

11 $${\lambda }_{\alpha }(\begin{array}{c}{p}_{\alpha }\\ {q}_{\alpha }\end{array})=(\begin{array}{cc}{f}_{S}+\varepsilon {\Lambda }_{\alpha } & {f}_{I}\\ {g}_{S} & {g}_{I}+\sigma \varepsilon {\Lambda }_{\alpha }\end{array})(\begin{array}{c}{p}_{\alpha }\\ {q}_{\alpha }\end{array}),$$

where *λ*_*α*_ denotes eigenvalues of the linearized system (2).

### Eigenvalues of the disease free equilibrium

When the Jacobian matrix of system (3) is evaluated at the disease free equilibrium, we will obtain *f*_*S*_ = −*r*, *f*_*I*_ = −*β*, *g*_*S*_ = 0, and (*β* − *γ*). To obtain stable endemic equilibrium, parameter *β* − *γ* should be negative. The eigenvalues of system (11) for each Λ_*α*_ are given by ${\lambda }_{\alpha }^{\mathrm{(1)}}=-\,r+\varepsilon {\Lambda }_{\alpha }$ and ${\lambda }_{\alpha }^{\mathrm{(2)}}=\beta -\gamma +\sigma \varepsilon {\Lambda }_{\alpha }$. The eigenvalues of the Laplacian matrix will be non-positive, i.e. Λ_*α*_ ≤ 0. One easily can see that eigenvalues of system (11) evaluated at the disease free equilibrium will be negative. Therefore, system (2) cannot admit pattern formations around the disease free equilibrium.
